# Facile Implementation of Antimicrobial Coatings through Adhesive Films (Wraps) Demonstrated with Cuprous Oxide Coatings

**DOI:** 10.3390/antibiotics12050920

**Published:** 2023-05-17

**Authors:** Saeed Behzadinasab, Myra D. Williams, Joseph O. Falkinham, William A. Ducker

**Affiliations:** 1 Department of Chemical Engineering, Virginia Tech, Blacksburg, VR 24061, USA; saeedb@vt.edu; 2 Center for Soft Matter and Biological Physics, Virginia Tech, Blacksburg, VR 24061, USA; 3 Macromolecules Innovation Institute, Virginia Tech, Blacksburg, VR 24061, USA; 4 Department of Biological Sciences, Virginia Tech, Blacksburg, VR 24061, USA

**Keywords:** antimicrobial, antibacterial, wrap, copper, cuprous oxide, Cu_2_O, coating, bacteria, polydopamine, polyurethane

## Abstract

Antimicrobial coatings have a finite lifetime because of wear, depletion of the active ingredient, or surface contamination that produces a barrier between the pathogen and the active ingredient. The limited lifetime means that facile replacement is important. Here, we describe a generic method for rapidly applying and reapplying antimicrobial coatings to common-touch surfaces. The method is to deposit an antimicrobial coating on a generic adhesive film (wrap), and then to attach that modified wrap to the common-touch surface. In this scenario, the adhesion of the wrap and antimicrobial efficacy are separated and can be optimized independently. We demonstrate the fabrication of two antimicrobial wraps, both using cuprous oxide (Cu_2_O) as the active ingredient. The first uses polyurethane (PU) as the polymeric binder and the second uses polydopamine (PDA). Our antimicrobial PU/Cu_2_O and PDA/Cu_2_O wraps, respectively, kill >99.98% and >99.82% of the human pathogen, *P. aeruginosa,* in only 10 min, and each of them kill >99.99% of the bacterium in 20 min. These antimicrobial wraps can be removed and replaced on the same object in <1 min with no tools. Wraps are already frequently used by consumers to coat drawers or cars for aesthetic or protective purposes.

## 1. Introduction

The human and economic burdens of infectious disease are vast [1,2,3,4,5]. Reducing the frequency of infections reduces this burden and reduces the demand for antibiotics and the concomitant danger of selection for antibiotic resistance in microorganisms [6,7,8]. Antibiotic resistance has worsened recently [9,10,11], and sadly, more than one million people die every year from antibiotic-resistant bacterial infections [8]. These infections occur both in healthcare settings [12] and in the general public domain. Infections occur via a number of routes, and here, we focus on transmission via contaminated solids, also known as fomite transmission [13,14].

The most common strategy to inhibit disease transmission via fomites is to disinfect solid surfaces by wiping them with bleach or 70% ethanol, but it is time-consuming to wipe many surfaces, and some disinfectants (e.g., bleach) are hazardous [15]. Perhaps most importantly, there is no protection subsequent to disinfection: the surface can be re-contaminated within minutes. An alternate approach is to provide ongoing protection with a surface coating that inactivates, or kills, pathogens for an extended period (e.g., weeks) after the coating is applied. Such coatings have shown effectiveness against a variety of pathogens [16,17,18,19,20,21,22,23,24,25,26,27]. Important aspects of antimicrobial coatings are that they do not use antibiotics and therefore are less likely to contribute to antibiotic resistance, and they operate outside the body. They provide an additional approach rather than an alternative measure against infection. Work by Ellingson et al. [28] has demonstrated the efficacy of antimicrobial surface coatings in a hospital setting; the coating caused significant reductions in both the number of bacteria detected on surfaces and the number of healthcare-acquired infections (HAI). Furthermore, Marik et al. showed that the use of copper oxide coatings on hard surfaces in a hospital was correlated with a reduction in infections [29].

Antimicrobial coatings can be either incorporated during the manufacture of an object or applied to the finished object. Application to existing facilities is particularly important for a rapid response to evolving situations such as the COVID-19 pandemic: it is expensive and time-consuming to replace many common-touch items (e.g., bed railings) in a short period of time. Ideally, the installation of antimicrobial coatings in existing facilities should be rapid and should not require sophisticated technical skills or equipment so that it can be carried out by non-experts on site.

In this manuscript, we describe a method for rapidly applying an antimicrobial coating to common-use objects. The method is simple. We prepare an antimicrobial coating with an adhesive backing layer that can be simply applied to a solid by removal of the backing paper, application to the solid, and sometimes followed by moderate pressure onto the solid. This type of coating is common for cars, where it is called a wrap, or for lining drawers, where it is called contact paper, and is available in sheets with dimensions of meters. The primary criteria for the preparation of the antimicrobial wrap are that application of the antimicrobial layer does not degrade the integrity of the wrap and does not reduce its ability to adhere to surfaces. The use of a wrap also solves an important problem with antimicrobial coatings. Antimicrobial coatings have a finite lifetime because of wear, depletion of the active ingredient, or surface contamination (e.g., oils from skin) that produces a barrier between the pathogen and the active ingredient. When the coating is no longer functional, the coating must be cleaned (to remove contamination) or removed and replaced with a functional coating. The removal step can be time-consuming as is well-known for repainting houses, etc. For situations where the coating must be replaced frequently, the time for replacement is a significant cost, whether or not the old coating must be removed. In situations such as these, a wrap is very efficient. When the old coating is compromised, the wrap is peeled off, and a new wrap is applied. The adhesive properties of the wrap can be optimized for easy removal or longevity, depending on the application. In general, the adhesion and the antimicrobial layer can be optimized independently.

This work describes two antimicrobial wraps that allow the very easy conversion of a common-touch surface into an antimicrobial common-touch surface. Both wraps utilize a potent antimicrobial material: cuprous oxide (Cu_2_O) [30,31]. Cu_2_O has broad antimicrobial activity against many pathogens, for example, against *Pseudomonas aeruginosa*, *Escherichia coli*, *Staphylococcus aureus,* and *Enterococcus hirae* [18,32,33], and methicillin-resistant *S. aureus* (MRSA) [18,19]. The wraps were coated with Cu_2_O using previously developed coatings [18,19,20] that were shown to rapidly inactivate a variety of pathogens and were robust to abrasion [19]. The other ingredients in these coatings were shown to be inactive.

We provide measurements of the bactericidal activity of the Cu_2_O wraps against *P. aeruginosa. P. aeruginosa* is labeled by the United States (US) Center for Disease Control (CDC) as a serious threat [34] because *P. aeruginosa* has developed antibiotic resistance to many antimicrobials [35,36]. Of particular relevance to this study, it remains viable [37] and forms biofilms on surfaces [38].

To illustrate the versatility, we prepared two antimicrobial coatings on the same commercial wrap, which was adhesive-backed polyethylene (PE). The first wrap was prepared using polyurethane (PU/Cu_2_O wrap), which caused a 99.97% reduction in *P. aeruginosa* colony-forming units (CFUs) in 10 min and >99.99% in 20 min. The second used polydopamine (PDA/Cu_2_O wrap) which caused a reduction of 99.80% in 10 min and >99.99% in 20 min. Polydopamine (PDA) has numerous advantages: it is derived from natural sources, uses water as the polymerization medium, has a high LD_50_ (~500 mg/kg), and polymerizes on most solid materials [39,40].

## 2. Results

### 2.1. Preparation and Characterization of the Antimicrobial Wraps

**Figure 1:**
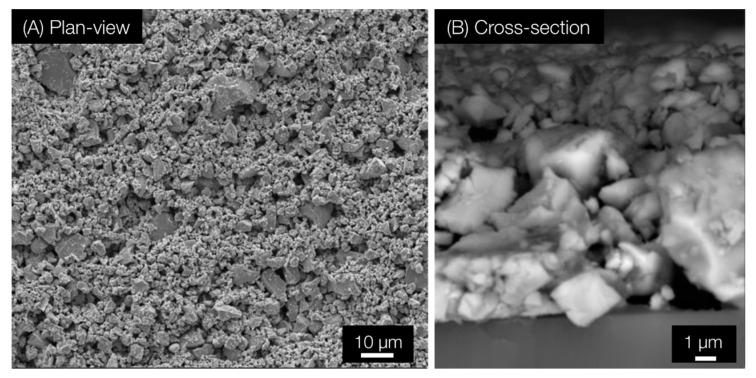
(**A**) Plan-view and (**B**) cross-section SEM images of PU/Cu_2_O wrap. The thickness of the coating was approximately 12 µm. For SEM imaging, the coating was prepared on glass.

The fabrication of both wraps is uncomplicated. The PU/Cu_2_O wrap was prepared in a two-step procedure where a PE wrap was first coated with polyurethane (PU), and then, a suspension of Cu_2_O in ethanol was cast on the tacky PU layer. The sample was heated for 2 h to accelerate the cure of PU. Scanning electron microscopy (SEM) images of the coating are presented in Figure 1. The PU/Cu_2_O coating only had a thickness of 10–20 µm, and the loading of particles in the PU/Cu_2_O wrap was 4.5 × 10^−3^ g/cm^2^. Because the coating is thin, only a very small amount of copper is used per area, which reduces the cost of implementation.

**Figure 2:**
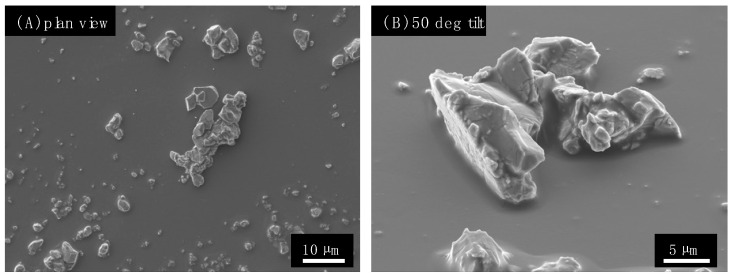
SEM images of PDA/Cu_2_O wrap. (**A**) Plan-view SEM image and (**B**) 50 degree tilt image. The coating was prepared on glass.

The PDA/Cu_2_O wrap was prepared by applying a suspension of Cu_2_O in dopamine to the uncoated wrap, and then, it was heat-treated to complete the polymer cure. Figure 2 shows SEM images of the PDA/Cu_2_O wrap. As shown in Figure 2B, the Cu_2_O particle morphology is visible, indicating that the particles protrude from the coating and therefore are accessible to solutions and bacterial suspensions. The particles form a much sparser layer on the solid compared to the PU/Cu_2_O wrap. The PDA/Cu_2_O wrap has a loading of 0.13 × 10^−3^ g/cm^2^ of Cu_2_O particles, which is about 1/35th of the loading of PU/Cu_2_O wrap, thereby enabling even lower costs of the active ingredient.

### 2.2. The Wraps Are Highly Antimicrobial

The antimicrobial activities of the wraps were tested by placing a 5 µL droplet of approximately 1.6 × 10^7^ CFU/mL *P. aeruginosa* on each wrap, waiting for a preset interval (0, 10, or 20 min), and extracting the bacteria from the solid, followed by counting the colonies formed by bacteria that remained viable (CFUs). The resulting CFUs were compared to the input CFUs to obtain the log survival, as defined by the following equation:

(1) $$log\;survival=mean\left[{log}_{10}\left(sample\;CFU\right)\right]-mean\left[{log}_{10}\left(input\;CFU\right)\right]$$

Both wraps caused a large decrease in the bacterial viability in a short period of time.

**Figure 3:**
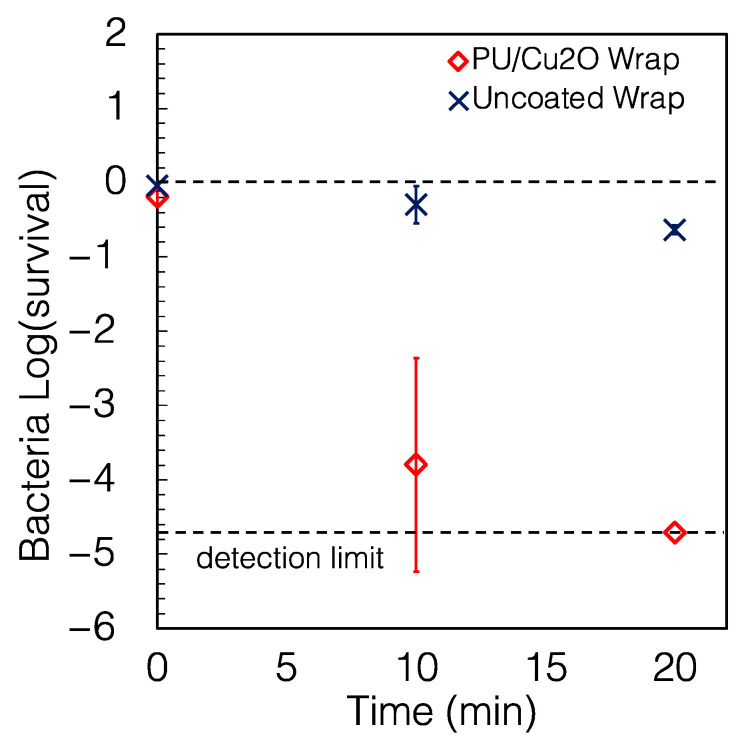
Survival of *P. aeruginosa* on PU/Cu_2_O wrap. The PU/Cu_2_O wrap caused a reduction of 99.97% and >99.99% of *P. aeruginosa* in 10 and 20 min, respectively, compared to the uncoated wrap. Survival was calculated using Equation (1). Each point in this and subsequent figures of survival represents the average of three independent measurements, and the error bars represent the standard deviation. Some error bars are smaller than the size of the symbols.

For the PU/Cu_2_O wrap, as shown in Figure 3, the log (CFU) decreased by 3.8 log units (99.984% killing) within 10 min, and no colonies were detected after 20 min (>99.998% killing), demonstrating excellent antimicrobial properties. The fitted half-life was 1.3 min (95% CI of 0.88–2.69 min). The half-life was calculated using Equation (2) in the Section 4. Bacteria can die on surfaces that do not have active ingredients, and for this reason, the US Environmental Protection Agency (EPA) [41,42] uses as its metric a comparison to the uncoated solid. For this reason, we also calculated “reduction” (see Equation (5)) in the Section 4) to compare the coated and uncoated wrap. The PU/Cu2O wrap caused a reduction of 99.97% of *P. aeruginosa* in 10 min, and >99.99% (4 log reduction) in 20 min. For registration, the EPA requires an antimicrobial coating to cause a minimum 3-log (99.9%) reduction in 1 h; the wrap achieved a greater reduction in only 10 min. Using the 99.9% reduction from the control as our metric, we performed Student’s *t*-tests separately on the 10 min and 20 min data, comparing 1/1000th of the control CFUs to the wrap CFUs to show that the antimicrobial wrap caused a reduction of >99.9% (*p* < 0.001) compared to the uncoated wrap after 20 min, but that this target was not achieved in 10 min (*p* = 0.22).

**Figure 4:**
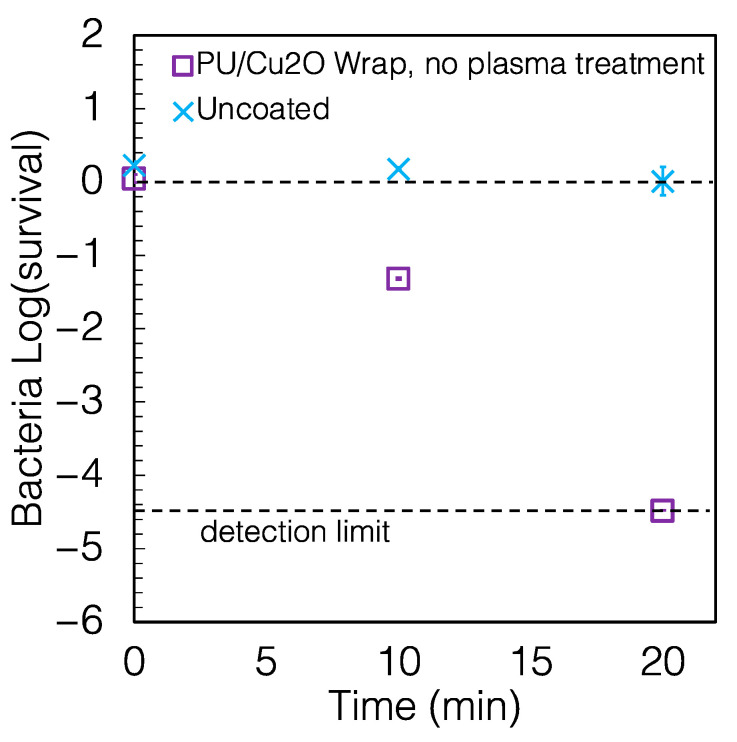
Survival of *P. aeruginosa* on PU/Cu_2_O wrap that was not heat-treated or plasma-treated. The wrap caused a reduction of 96.81% and >99.99% of *P. aeruginosa* in 10 and 20 min, respectively. Some error bars are smaller than the size of the symbols.

The preparation of the PU/Cu_2_O coating described above included heat and plasma treatments, whereas for simplicity, it would be beneficial to prepare a coating that does not require these steps. We prepared a second set of PU/Cu_2_O coatings with no heat or plasma treatment. As shown in Figure 4, these wraps killed 95.177% of bacteria in 10 min and >99.996% in 20 min, i.e., a slightly reduced performance than the wrap with the extra treatments at 10 min but no resolved difference at 20 min. The reduction compared to the uncoated control was again >99.9% (*p* = 0.003) for 20 min of exposure.

**Figure 5:**
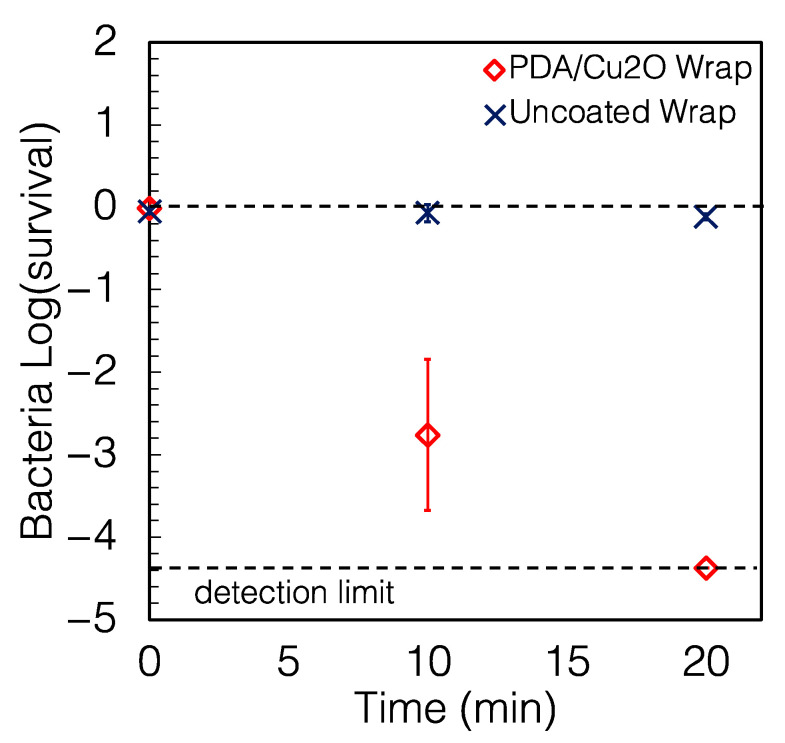
Survival of *P. aeruginosa* on PDA/Cu_2_O wrap. The PDA/Cu_2_O wrap caused a reduction of 99.80% and >99.99% of *P. aeruginosa* in 10 and 20 min, respectively. The reduction compared to the uncoated wrap was >99.9% (*p* < 0.001) again for 20 min exposure.

The PDA/Cu_2_O wrap was also extremely effective against *P. aeruginosa*, as shown in Figure 5. The wrap killed 99.826% in 10 min and >99.995% in 20 min and had a half-life of 1.38 min (95% CI of 1.10–1.85 min). It caused a reduction of 99.80% and >99.99% of the bacterium in 10 and 20 min, respectively. It is important to note that this very similar level of performance was achieved with about 1/35th of the loading of the PU/Cu_2_O wrap. This shows that the Cu_2_O is a very potent antimicrobial, and it is unnecessary to provide the high loading of the PU/Cu_2_O wrap to achieve a good performance in these tests.

### 2.3. The Antimicrobial Wraps Are Easy to Replace

**Figure 6:**
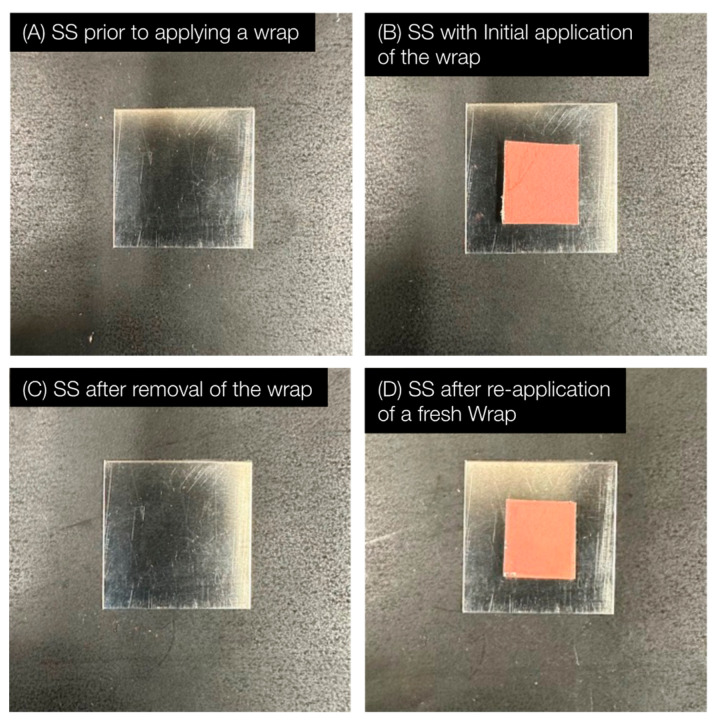
Series of photographs demonstrating the ability to apply, remove, and reapply an antimicrobial wrap to stainless steel (SS). (**A**) Bare SS. (**B**) Initial application of antimicrobial wrap to SS. (**C**) After removal of wrap. (**D**) After application of a new antimicrobial wrap to the same SS. Removal and replacement of the wraps took less than one minute.

Recall that the motivation for this work is the simple replacement of an antimicrobial coating to overcome the problems of wear and contamination. Both the PU/Cu_2_O and PDA/Cu_2_O wraps were designed for rapid replacement. Figure 6 is a series of photographs where a PU/Cu_2_O wrap was added and then replaced. The replacement of the coating took less than 1 min and was achieved without tools.

### 2.4. Other Characteristics of the Coating

**Figure 7:**
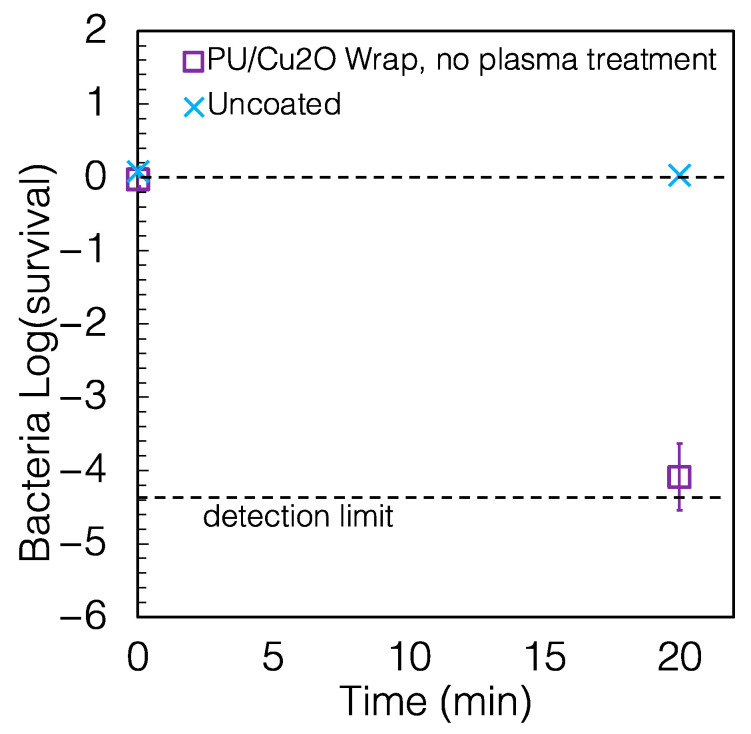
Survival of *P. aeruginosa* on an aged PU/Cu_2_O wrap (no heat or plasma treatments). The antimicrobial wrap was 3.5 months old at the time it was tested. The aged wrap caused a reduction of >99.99% of *P. aeruginosa* in 20 min. Some error bars are smaller than the size of the symbols.

Here, we discuss characteristics of the specific coatings that we used on the wraps. The PU/Cu_2_O coating used ethanol, which is a relatively safe solvent. The polymerization of dopamine only requires water as the solvent. Both coatings require a low temperature to complete the cure (80 or 120 °C), and we found that heating was not even required for the PU coating. In any case, this heat treatment is not required on site but is only used for manufacturing. Paintable PU formulations have some toxicity due to the evaporation of solvents, but this need only be carried out during the manufacture of the wrap, not at the implementation site. The Median Lethal Dose (LD_50_) of polydopamine is high, about 500 mg/kg [43]. Cuprous oxide is an abundant material made from recycled copper and costs only a few dollars per kg. Additionally, other materials used in this study are inexpensive, which is beneficial for implementation. Furthermore, the coatings remain potent after a long time. For example, as seen in Figure 7, 3.5-month-old PU/Cu_2_O wrap (no plasma or heat-treatment) that was tested against *P. aeruginosa* remained active against the bacterium. Finally, the processes used to prepare both antimicrobial wraps do not require complex equipment.

## 3. Conclusions

The utilization of antimicrobial coatings has been shown to correlate with a lower number of infections [28,29]. However, it can be a significant challenge for an untrained person to install antimicrobial coatings in their desired location. Here, we show that a simple adhesive-backed version of an antimicrobial coating could be fabricated and that it retained antimicrobial efficacy for 3.5 months. This antimicrobial coating can be applied in the field in seconds, as demonstrated on stainless steel. This is a very important feature for antimicrobial coatings that can become fouled or worn or whose active material can become depleted. Large area wraps have already been used on cars, and antimicrobial coatings can be sprayed or painted, so large- or small-scale objects can be made antimicrobial.

For the highest effectiveness, an antimicrobial wrap needs to kill microbes rapidly. We prepared two different antimicrobial wraps based on the active ingredient Cu_2_O. One utilized polyurethane and the other polydopamine as the binding polymer. Both coatings killed >99.995% of *P. aeruginosa* in 20 min, and the bacteria had half-lives of <1.4 min. By demonstrating the preparation of two antimicrobial coatings on the same base wrap, we showed that the wrap concept is modular, which allows the independent optimization of the adhesive coating and the antimicrobial surface.

## 4. Materials and Methods

### 4.1. Materials

In this study, 70% ethanol was purchased from VWR (Radnor, PA, USA). Dopamine hydrochloride (99%) was obtained from Fisher Scientific (Waltham, MA, USA). In-house DI water was further purified using a Milli-Q Reference system. Tris(hydroxymethyl)aminomethane (Tris, ACS reagent) was obtained from Sigma Aldrich. Adhesive-backed polyethylene sheets (catalog number: 8722K23) and stainless steel 301 were purchased from McMaster-Carr (Elmhurst, IL, USA), washed with (70%) ethanol, rinsed with DI water, and dried with nitrogen gas. Cu_2_O particles (Chem Copp HP III UltraFine Type-5; mean size = 5.4 µm) were obtained from American Chemet Corporation (Deerfield, IL, USA). Polyurethane (PU; Miniwax, Fast-Drying Polyurethane, clear satin) was obtained from Lowes Home Improvement departmental store (Williamsburg, VA, USA). TSA (Tryptic Soy Agar) and TSB (Tryptic soy broth) were purchased from BD, Sparks, MD, USA.

### 4.2. Fabrication of Antimicrobial PU/Cu_2_O Wrap

A PU/Cu_2_O coating was applied as described previously [20]. In brief, a thin layer of PU was cast onto the uncoated wrap and air-dried for a few minutes. A suspension of 10% Cu_2_O in ethanol was subsequently applied on the PU film, and it was left to partially dry for a few min. The coated wrap was then heat-treated in an oven (120 °C for 2 h) to speed up the formation of the PU coating, washed using DI water, and cut into smaller pieces (12 × 12 mm). In some cases, where indicated, the wrap was plasma-treated with Argon (3 min, at 100 W, and <200 mTorr). The antimicrobial wrap was applied to stainless steel.

### 4.3. Fabrication of Antimicrobial PDA/Cu_2_O Wrap

The PDA/Cu_2_O wrap was prepared using the method in reference [19]. A suspension of Cu_2_O particles (0.2% *w*/*w*) in 10 mm tris solution was sonicated, and dopamine hydrochloride was added to the suspension to yield a final concentration of 0.05 g dopamine/L. Subsequently, 140 µL of the prepared suspension was applied to the uncoated wrap (15 × 15 mm in size). The sample was heat-treated in an oven (80 °C for 30 min). The antimicrobial wrap was applied to stainless steel.

### 4.4. Characterization of Test Solids

The morphology of the coatings was characterized via scanning electron microscopy (SEM) using either a JEOL IT500 SEM (Tokyo, Japan) or a FEI Quanta 600 FE-ESEM (Telč, Czech Republic). Samples for SEM were treated with a 5 nm layer of Pt/Pd to enhance electrical conductivity.

### 4.5. Antibacterial Assay

#### 4.5.1. Choice of Microbial Strain

According to the United States Environmental Protection Agency [41,42] guidance, *P. aeruginosa* (strain DSM-9644) was chosen to test the antimicrobial properties of the samples.

#### 4.5.2. Growth of Microbial Strains

Bacteria were grown to the mid-exponential phase at 37 °C in 5 mL of Tryptic Soy Broth (TSB) with aeration (rpm = 60). For the visual verification of the identity and purity of the bacterium, cells were streaked from the liquid culture on Tryptic Soy Agar (TSA) plates and then incubated (37 °C for 48 h). The specific color and morphology traits of *P. aeruginosa* were confirmed before using the batch to test the antimicrobial coatings.

#### 4.5.3. Preparation of Microbial Strains for Testing

Bacterial cells were extracted from liquid cultures via centrifugation (20 min at 5000× *g*), followed by the removal of the supernatant and then re-suspension in 5 mL of Phosphate-Buffered Saline (PBS), followed by vortexing (60 s). Two rounds of centrifugation and resuspension in PBS were carried out.

#### 4.5.4. Measurement of Viable Cell Number

Viable cell number was determined from colony forming units (CFUs). CFU/mL of the suspension was measured by spreading 0.1 mL of a suspension on a TSA plate (in triplicate), and colonies were counted after incubation for 48 h at 37 °C. Because of the large range of CFU, each test suspension was used to create a 10-fold dilution series, and each of these dilutions were measured to obtain the optimum number of CFUs for counting (20–200). The suspensions had a CFU/mL of approximately 1.6 × 10^7^. If zero colonies were present for the lowest dilution, that number was counted as one colony to enable a log_10_ transformation of all the data. This was our detection limit for measurement on a particular plate.

#### 4.5.5. Measurement of Surface-Killing

A 5 µL droplet of *P. aeruginosa* cells in PBS was placed on solid test samples six days after the antimicrobial wraps were prepared. An uncoated wrap was used as the control. After a known time interval (e.g., 0, 10, or 20 min), an individual sample was placed in a 50 mL centrifuge tube containing 5 mL of PBS. The sample was then vortexed (10 s and at the highest setting) and sonicated (1 min; model: Branson Model 12 Ultrasonic Cleaner, Shelton, CT, USA) to detach all cells from the solid. Assuming the resulting suspension of cells was homogeneous, the total number of surviving cells could be measured by obtaining the CFU/mL of this bacterial suspension. In total, 0.1 mL of the suspension was removed without contacting the sample, and the CFUs were measured as described above. In addition, the CFU/mL for input bacterial suspension was measured for the calculation of survival. Three independent solid samples were tested in each condition (i.e., coated or uncoated at each time point), and three repeats were carried out for each of these independent measurements. The detection limit and resolution on the agar plate was 1 colony, but because only 0.1 mL of the 5 mL volume was sampled, the detection limit of bacteria was 50 CFU or log (CFU) = 1.7.

### 4.6. Statistical Analysis

All statistics were calculated from log CFUs, and Student’s *t*-tests were two-tailed and heteroscedastic. A significance level of 0.05 was employed for statistical analysis. The half-life was calculated from a linear fit of the slope of all log (CFU)–time data at the three time points. The half-life is:

(2) $$t_{1/2}=-\frac{0.30}{slope}$$

All data from 0 to 20 min were used to calculate $t_{1/2}$, and the uncertainty was the 95% confidence interval (CI). Because the CFUs at 20 min were all below the detection limit, the calculated half-life was an upper bound.

### 4.7. Calculation of Microbial Survival and Reduction

The CFU counting technique counts cells that can reproduce enough times to form a visible bacterial colony. We use the term “kill” as a shorthand for cells that cannot form a colony. Percentage kill is a comparison of the CFUs measured after a specific time to the CFUs of the input suspension:

(3) $$log\;kill=mean\left[{log}_{10}\left(input\;CFU\right)\right]-mean\left[{log}_{10}\left(sample\;CFU\right)\right]$$

(4) $$\%\;kill=\left[1-10^{-log\;kill}\right]\times 100$$

For calculations, the measured sample CFUs were adjusted for dilutions that took place during extraction of bacteria from the solid. When compared to a standard at the same time, we used reduction, defined as:

(5) $$log\;Reduction=mean\left[{log}_{10}\left(uncoated\;sample\;CFU\right)\right]\;-mean\left[{log}_{10}\left(antimicrobial\;sample\;CFU\right)\right]$$

(6) $$\%\;Reduction=\left[1-10^{-log\;Reduction}\right]\times 100$$

where the same volume and concentration of bacterial suspension was added to the coated and uncoated sample. In this paper, the uncoated sample was always the uncoated commercial wrap.

We began the statistical treatment with a log transformation because we found that the residuals were approximately normally distributed after a log transformation. Each CFU measurement in Equations (3) and (5) is the mean of three repeat counts of a different agar plate from the liquid sample; each mean in Equations (3) and (5) is the mean from three measurements on three independent samples. *p*-values for 99.9% reduction were obtained by the following method: for each of the three independent measurements, we calculated:

$$\left[{log}_{10}\left(uncoated\;sample\;CFU\right)\right]-3$$

and then performed a one-sided *t-*test to determine whether those values were different to the three independent values of $\left[{log}_{10}\left(antimicrobial\;sample\;CFU\right)\right]$ for the same time condition. The resulting *p*-value indicated the confidence that the antimicrobial coating decreased the cell numbers by more than 99.9%.

## Data Availability

Data will be provided on request.
